# Analysis of optical absorption in GaAs nanowire arrays

**DOI:** 10.1186/1556-276X-6-617

**Published:** 2011-12-06

**Authors:** Haomin Guo, Long Wen, Xinhua Li, Zhifei Zhao, Yuqi Wang

**Affiliations:** 1 Key Laboratory of Materials Physics, Institute of Solid State Physics, Chinese Academy of Sciences, Hefei, 230031, People's Republic of China

## Abstract

In this study, the influence of the geometric parameters on the optical absorption of gallium arsenide [GaAs] nanowire arrays [NWAs] has been systematically analyzed using finite-difference time-domain simulations. The calculations reveal that the optical absorption is sensitive to the geometric parameters such as diameter [*D*], length [*L*], and filling ratio [*D/P*], and more efficient light absorption can be obtained in GaAs NWAs than in thin films with the same thickness due to the combined effects of intrinsic antireflection and efficient excitation of resonant modes. Optimized geometric parameters are obtained as follows: *D *= 180 nm, *L *= 2 μm, and *D/P *= 0.5. Meanwhile, the simulation on the absorption of GaAs NWAs for oblique incidence has also been carried out. The underlying physics is discussed in this work.

**PACS: **81.07.Gf nanowires; 81.05.Ea III-V semiconductors; 88.40.hj efficiency and performance of solar cells; 73.50.Pz photoconduction and photovoltaic effects.

## Background

Semiconductor nanowire arrays [NWAs] are presently under intense research and development for next-generation solar cells due to their potential for lower cost and greater energy conversion efficiency compared to conventional thin film devices [1-4]. Among semiconductor nanowires [NWs], gallium arsenide [GaAs] NWs show particular promise due to the superior electrical and optical properties of III to V materials. For example, the GaAs material system features a direct band gap and high absorption coefficient. This makes GaAs NWs prime candidates for future optoelectronic devices, just as bulk materials [5-7]. Recently, many advances have been reported in the fabrication of GaAs NW solar cells. For example, Czaban et al. observed a photovoltaic [PV] effect with a photoconversion efficiency of 0.83% from vertically oriented GaAs NWs grown on n-GaAs(111)B substrates [5]. Colombo et al. reported a coaxial p-i-n single nanowire cell with an efficiency of 4.5% [7]. These results illustrate that the efficiency of GaAs NW PV devices is much lower than that of thin film counterparts. There are still many problems to be resolved before GaAs NWs can become available for practical applications.

One of the main issues on nanowire solar cells is the determination of the nanowire geometry. It has been proved by many theoretical and experimental works that NWAs with well-defined geometric parameters such as diameter, length, and filling ratio exhibit a much more efficient light absorption in the solar spectrum [1-4,8-11]. In this paper, the influence of geometric parameters on the optical absorption in GaAs NWAs is analyzed using finite-difference time-domain [FDTD] simulations [12]. Optimized geometric parameters are obtained through the simulations. The underlying physics is discussed in this work.

## Results and discussion

**Figure 1:**
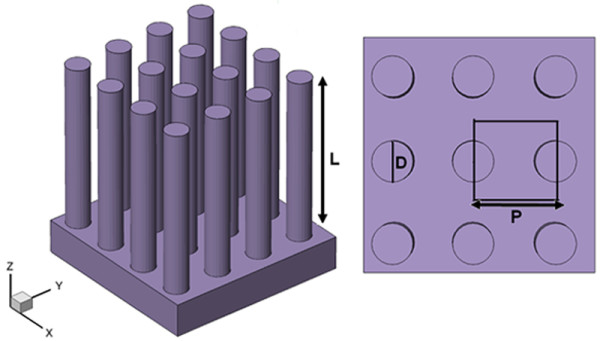
**Schematic drawing of the periodic GaAs NWAs structure**.

Figure 1a illustrates the vertically aligned GaAs NWAs structure we study. The array consists of GaAs NWs with diameter *D*, period *P*, and wire length *L *arranged in a square lattice and surrounded by air. The thickness of the underlying GaAs thin film substrate is set to 200 nm. The wavelength-dependent complex refractive index used to describe the material dispersion properties of GaAs can be obtained from the study of Levinshtein et al [13]. By applying periodic boundary conditions in the *x *and *y *directions, the simulations are carried out within this unit cell to model the periodic NWAs structure. The simulation domain is closed at the top and bottom with a perfectly matched layer, allowing reflected light to escape the simulation volume. The incident light is firstly set in parallel to the NWs axis, and we use a plane wave with a wavelength ranging from 300 nm to 880 nm (typical absorption region of GaAs) to model the sunlight. Two power monitors are used in our simulations. The reflection monitor is located at 2 μm above the top surface of the NWAs, while the transmission monitor is located at bottom surface of substrate. In each simulation, the integrated Poynting power flow past the reflection and transmission monitor is recorded. Specifically, the amount of power transmitted through power monitors is normalized to the source power at each wavelength. The reflectance *R*(*λ*) and transmission *T*(*λ*) are calculated by the equation:

$$R\left(\lambda \right),T\left(\lambda \right)=0.5\int \text{real}\left\{p{\left(\lambda \right)}_{\text{monitor}}\right\}dS∕P_{\text{in}}\left(\lambda \right),$$

where *p*(*λ*) is the Poynting vector, *dS *is the surface normal, and *P*_in_(*λ*) is the incident source power at each wavelength. The absorption spectrum *A*(*λ*) of the GaAs NWAs is given by the equation:

$$A\left(\lambda \right)\;=\text{ 1}-R\left(\lambda \right)-T\left(\lambda \right).$$

**Figure 2:**
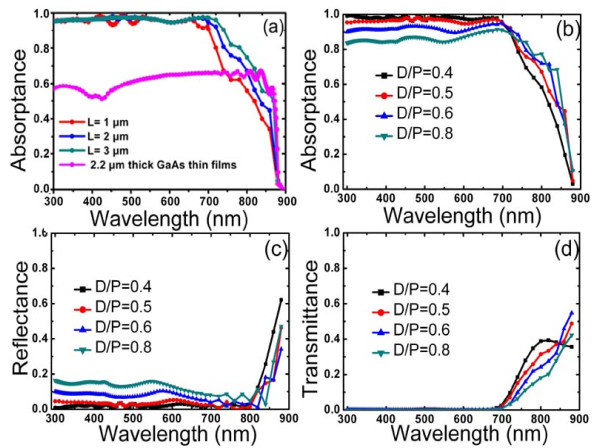
**Absorptance, reflectance, and transmittance of GaAs NWs**. (**a**) Absorptance of GaAs NWs with different lengths of 1 to 3 μm. The pink curve shows the absorptance of a 2.2-μm-thick GaAs thin film. (**b**, **c**, **d**) Absorptance, reflectance, and transmittance of GaAs NWAs with different filling ratios varying from 0.4 to 0.8.

Figure 2a shows the calculated absorptance of GaAs NWAs with different lengths (*D *= 180 nm, *D/P *= 0.5). Four lengths, 0.5, 1, 2, and 3 μm, are selected to calculate the thickness-dependent absorption. The absorptance of a 2.2-μm-thick GaAs film is also plotted in the figure for comparison. As seen in this figure, a sharp drop to zero absorption occurs for photon energies smaller than the corresponding bandgap. In short wavelengths, the absorptance spectra for all NWAs are maintained above 90%, which is much higher than those of the thin film. The absorptance enhancement in NWAs is mainly attributed to the lowered reflectance at the top surface of NWAs. The effective refractive index of the NWAs can be approximated using a volume average:

$$n_{\text{eff}}=\;n_{\text{air}}\left(1-f\right)+\;n_{\text{GaAs}}f,$$

where *f *= *πD^2^/4P^2 ^*and *n*_air _and *n*_GaAs _are the refractive indexes of air and GaAs, respectively. Therefore, the effective refractive index of the NWA is much lower than that of the thin film counterparts, resulting in a perfect refractive index matching at the top interface between the air and NWAs, hence leads to good coupling of the incident light into the NWAs [14-16]. In long wavelength region, the results clearly indicate that longer NWs have higher absorptance due to the increased optical path length in the NWAs. For photovoltaic device applications, however, it should be noted that longer NWs would sacrifice efficient carrier extraction properties and lead to unnecessary material consumption. Hence, in the following simulations, we fixed the length of the NWs to *L *= 2 μm.

Figure 2b, d compares the reflectance, transmittance, and absorptance of NWAs with *D/P = *0.4, 0.5, 0.6, and 0.8 for a fixed diameter of 180 nm. The calculated spectra reveal that the absorption is uniquely determined by the reflection and decreases with larger filling ratios in the visible wavelength region (*λ *< 700 nm). As seen from Figure 2d, only zero-order transmission exists owing to the high extinction coefficient of GaAs in these wavelengths. The trend of enhanced reflectance with the increased *D/P *as indicated in Figure 2c can be attributed to the heightened effective refractive index of the NWAs. In the long wavelength region, however, NWAs would undergo a significant transmission and reflection loss. The absorptance curve, shown in Figure 2b, tends to shift towards larger wavelengths as the filling ratio is increased. From these results, it can be concluded that the optimal filling ratio is determined by the trade-off between the reflection enhancement and light transmission suppression with the increase of *D/P*.

**Figure 3:**
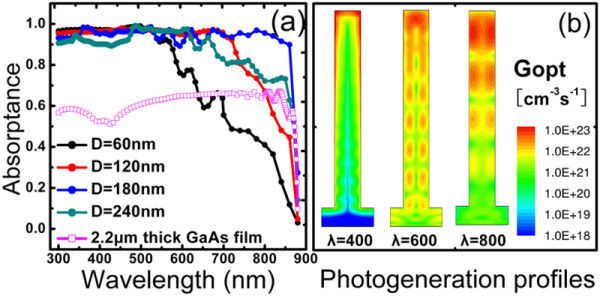
**Absorptance and photogeneration profiles**. (**a**) Absorptance of GaAs NWAs with different diameters varied from 60 to 240 nm. (**b**) Photogeneration profiles calculated at different wavelengths of 400, 600, and 800 nm by FDTD simulations.

To study the effect of diameters on the optical properties of NWAs, the absorption spectra for different arrays were calculated with a fixed *D/P *of 0.5 and diameters from 60 to 240 nm. As seen in Figure 3a, the light absorption is significantly enhanced by the increase of NW diameters from 60 to 180 nm in the long wavelength region. Similar results are also observed in silicon NWAs, which can be explained by the presence of a photonic resonance mode [14,15]. For the large refractive index contrast between the NWs and air, the electromagnetic field can be coupled efficiently into the NWs at resonances, resulting in a significant light-trapping ability boost. Due to the cylindrical geometry, the NW internal resonance is primarily determined by the diameters, but not by lengths. For GaAs NWAs with small diameters (approximately 120 nm or even less), most of the incident light cannot be coupled into the NWs, but is absorbed in a single path through the NWAs [16]. In comparison, NWs with larger diameters provide more supported modes; hence, the absorption is greatly increased when these modes are well coupled and concentrated within the NWs. As shown in Figure 2a, the absorptance values of NWAs with *D *= 180 nm remain above 90% across the entire above-bandgap wavelengths. However, a further increase in the wire diameter up to 240 nm leads to a decrease in absorption efficiency due to the enhanced light reflection at the top surface of the NWAs and the insufficient field concentration at long wavelengths.

To further illustrate the optical absorption of GaAs NWs, the optical generation rate, *G*_opt_, in a single nanowire throughout the simulation domain was calculated at each wavelength according to the equation:

$$G_{\text{opt}}=\frac{real\left(\nabla ∙\vec{p}\right)}{2\hbar \omega }=\frac{\varepsilon^{\prime \prime }|\overset{⇀}{E}{|}^{2}}{2\hbar },$$

where *ε″ *is the imaginary part of the permittivity and *E *is the electric field intensity. Figure 3b shows the cross-sectional distribution of the optical generation rate in a single nanowire for a same incident wave power of 100 mW/cm^2 ^with different wavelengths (*λ *= 400, 600, 800 nm). The optical generation rate for small wavelengths (e.g., 400 nm) is concentrated near the top and sides of the nanowire due to the strong wire-wire light scattering and short absorption length of GaAs at these photon energies. However, the generation rates for most of the solar spectrum (e.g., 600 and 800 nm) are concentrated near the core, demonstrating the internal absorption enhancement mode in the nanowire. Each nanowire acts as a nanoscale cylindrical resonator, which can trap light by multiple total internal reflections.

**Figure 4:**
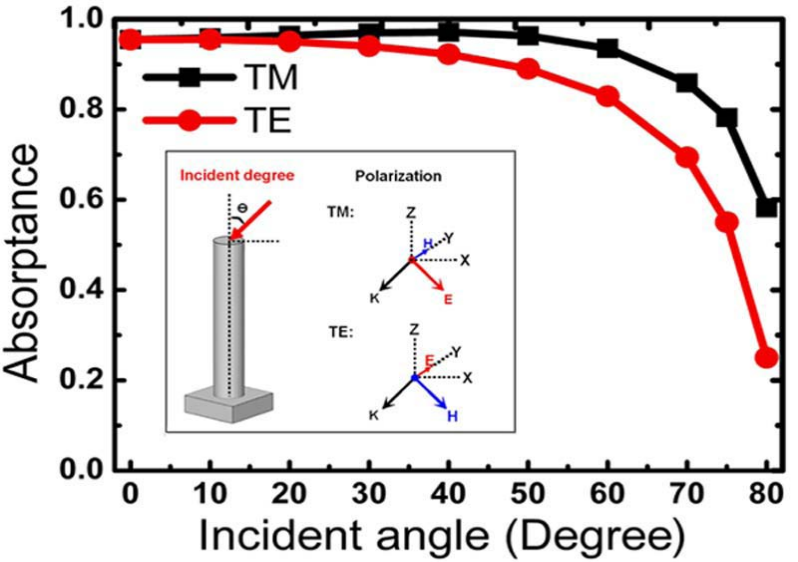
**Simulated absorptance of GaAs NWAs with optimized structure (*D/P *= 0.5, *D *= 180 nm)**. The absorptance was taken under oblique incident illumination at wavelength *λ *= 800 nm for different incident angles. The inset shows the schematic drawing of a single nanowire under oblique incident illumination together with definitions of TE and TM illumination.

The optical absorption of GaAs NWAs for oblique light incidence was also calculated with the optimized structure (*D*/P = 0.5, *D *= 180 nm) obtained for normal incident illumination. Figure 4 shows the absorptance of optimized NWAs at wavelength *λ *= 800 nm for different incident angles. The inset depicts the schematic drawing of a single nanowire under oblique incident illumination together with definitions of transverse-electric [TE] and transverse-magnetic [TM] illumination. At angles of incidence up to 60°, the total absorption is maintained above 80% for both TE and TM polarizations. This implies good antireflection properties for the coupling of oblique incident light into the NWAs. When there is a further increase in the angle of incidence beyond 60°, the absorption starts to decrease dramatically as less light can enter the medium because of the large reflectance. Meanwhile, a higher absorption is observed in TM polarization than in TE polarization, which implies the strong polarization anisotropy. This is due to the electric field component along the axis of TM polarization which would facilitate the absorption of the nanowires.

## Conclusions

In summary, we have analyzed the optical properties of GaAs NWAs by FDTD simulations, which were found to be sensitive to the structural parameters such as wire diameter *D*, length *L*, and filling ratio *D/P*. The optimal results for the normal incidence are evaluated as *D *= 180 nm, *L *= 2 μm, and *D/P *= 0.5. Our calculation shows that the absorptance exceeds 90% in well-designed GaAs NWAs in the visible light region, which is much higher than that of thin films with the same thickness due to the combined effects of the intrinsic antireflection and efficient excitation of resonant modes. The simulated optical generation rates in a single GaAs nanowire for most of the solar spectrum are concentrated near the core, illustrating the internal wire absorption enhancement mode. For the oblique incidence, perfect antireflection properties for the coupling of oblique incident light into the NWAs are demonstrated at incident angles up to 60°, while the absorption declines as the incident angle is over 60° due to the large reflectance. Meanwhile, a higher absorption is observed in TM polarization than in TE polarization, which is attributed to the electric field component along the axis of TM polarization.

## Competing interests

The authors declare that they have no competing interests.

## Authors' contributions

HMG and LW carried out the simulation of optical absorptance in the GaAs NWs and performed data analysis. XHL supervised the research in this study and took part in the discussions of the calculation results. ZFZ and YQW took part in the discussion of the underlying physics behind the results. All authors read and approved the final manuscript.
